# Barriers to global health development: An international quantitative survey

**DOI:** 10.1371/journal.pone.0184846

**Published:** 2017-10-03

**Authors:** Bahr Weiss, Amie Alley Pollack

**Affiliations:** Department of Psychology and Human Development, Vanderbilt University, Nashville, Tennessee, United States of America; Istituto Di Ricerche Farmacologiche Mario Negri, ITALY

## Abstract

**Background:**

Global health’s goal of reducing low-and-middle-income country versus high-income country health disparities faces complex challenges. Although there have been discussions of barriers, there has not been a broad-based, quantitative survey of such barriers.

**Methods:**

432 global health professionals were invited via email to participate in an online survey, with 268 (62%) participating. The survey assessed participants’ (A) demographic and global health background, (B) perceptions regarding 66 barriers’ seriousness, (C) detailed ratings of barriers designated most serious, (D) potential solutions.

**Results:**

Thirty-four (of 66) barriers were seen as moderately or more serious, highlighting the widespread, significant challenges global health development faces. Perceived barrier seriousness differed significantly across domains: *Resource Limitations* mean = 2.47 (0–4 Likert scale), *Priority Selection* mean = 2.20, *Corruption*, *Lack of Competence* mean = 1.87, *Social and Cultural Barriers* mean = 1.68. Some system-level predictors showed significant but relatively limited relations. For instance, for Global Health Domain, HIV and Mental Health had higher levels of perceived *Social and Cultural Barriers* than other GH Domains. Individual–level global health experience predictors had small but significant effects, with seriousness of (a) *Corruption*, *Lack of Competence*, and (b) *Priority Selection* barriers positively correlated with respondents’ level of *LMIC-oriented* (e.g., weeks/year spent in LMIC) but *Academic Global Health Achievement* (e.g., number of global health publications) negatively correlated with overall barrier seriousness.

**Conclusions:**

That comparatively few system-level predictors (e.g., Organization Type) were significant suggests these barriers may be relatively fundamental at the system-level. Individual-level and system-level effects do have policy implications; e.g., *Priority Selection* barriers were among the most serious, yet effects on seriousness of how LMIC-oriented a professional was versus level of academic global health achievement ran in opposite directions, suggesting increased discussion of priorities between LMIC-based and other professionals may be useful. It is hoped the 22 suggested solutions will provide useful ideas for addressing global health barriers.

## Introduction

The goal of the global health (GH) movement over the past two decades has been to reduce health disparities between high income countries (HIC), and low and middle income countries (LMIC) [1]. It is a complex task requiring coordination within and between countries of national, foreign national, and multi-national organizations, across numerous health care domains and professional disciplines. In general progress has been slower than hoped, and there is increasing awareness and recognition in the field of significant barriers that are proving challenging to address. For instance, Yamey [2] interviewed 14 implementation science experts heavily involved in global health, identifying a number of major barriers such as inadequate integration of research findings into scale-up efforts. He concluded that successfully scaling up of interventions in LMIC is a complex process involving a highly complex array of factors that faces many multifaceted barriers.

Overall, research regarding such barriers to global health development has focused on (A) the authors’ or a relatively small set of experts’ experiences regarding global health barriers, or (B) larger data-based studies of (B1) specific diseases, (B2) specific countries or geographical regions, or (B3) specific challenges to health development. Kim et al [3], for instance, discussed barriers to efforts to address serious health illness and global poverty, and based on their own experience proposed a framework for health-care delivery and evaluation. Other authors’ have discussed their own experiences of barriers for a particular disease or health focus (e.g., barriers to increased use of rapid diagnostic tests [4]; barriers to access to medications for non-communicable diseases [5]). A relatively limited number of articles have presented broader, more data-based analyses of barriers, important for maximizing the generalizability of plans for addressing barriers. Lim and Ojo [6], for example, conducted a systematic literature review of the utilization of cervical cancer screening in Sub-Saharan Africa, and identified several important barriers including cultural beliefs and practices, and negative experiences with health care. A third type of analysis has examined broader, more systemic barriers to health care development but within a relatively limited organizational focus. Gopinathan et al [7], for instance, interviewed senior WHO staff to assist the organization improve cross-sectorial collaboration and concluded in part that the WHO’s cross-sector involvement is hampered internally by over-emphasis on a biomedical perspective on health.

These studies provide important information regarding experts’ perceptions of GH development barriers, and about barriers for specific health concerns, health sectors, geographic regions, and agencies. There has not yet been, however, a more broad-based, international quantitative survey of GH professionals’ perceptions of GH barriers. Relatively little is known about how perceptions of barriers differ across geographic regions, health domains, and types of GH organizations. As Kim et al [3] and other authors have noted, it is becoming increasingly clear that a multi-level systems approach will be essential for most effectively and efficiently addressing global health barriers and for policy and planning purposes. This will require understanding of how perceptions of barriers vary across different health domains, organizations, and geographic regions. For instance, if perceptions of barriers vary across different types of organizations, this would suggest that there may be some level of lack of trans-agency coordination and reduced efficiency in approaching barriers, and understanding where and for which barriers this occurs will be critical. Similarly, although there is a clear need to tailor and individualize programs and approaches for different regions [8], at the international agency level at least there must be coordination and collaboration across regions to maximize efficiency, so understanding regional, etc. differences in perspectives is essential.

Relatively little also is known about the factors that may underlie perceptions of barrier severity, such as the effects of different barriers on the development versus sustainability of effective health programs. Such understanding is critical in order to ensure that the full impact of the barriers is considered in their solutions. Weiss et al [9] have described a model for sustainable global health development that may be useful for this purpose. In this model, there are twin *Capacity Development Goals*: Program Efficacy, and Program Sustainability, explicitly defined to support long-term development success. *Capacity Development Targets*, such as development of local research capacity, and long-term training of trainers (e.g., through university degree-granting programs [10]) serve to move capacity development towards the effectiveness and the sustainability goals (e.g., development of research capacity allows for objective assessment of program effectiveness; development of long-term trainers supports sustainability). Understanding the extent to which GH professionals consider these and other relevant factors in their perceptions of barriers will be important in order to ensure that the most effective, efficient, and sustainable solutions are developed.

The purpose of the present study was to conduct a quantitative, broad-based, international survey of global health professionals’ perceptions of barriers to GH development. The study focused on survey respondents’ perceptions of the seriousness of different barriers. For the reasons noted above, it assessed the extent to which perceived barrier seriousness varied as a function of system-level factors, including geographic region, global health focus, and the type of organization under which the respondent worked. In addition, it assessed the extent to which barrier seriousness varied as a function of individual-level factors related to respondents’ experience and orientation in the global health field. Most prior studies in this area (e.g., Yamey [2]) have relied on the perspective of ‘leaders’ in global health (i.e., people with high levels of experience and broad international recognition), but we purposely sampled professionals with varying ranges of experience in order to determine how perceptions differ as a function of these factors. We predicted that perceptions of barrier seriousness would vary as a function of these various variables, although because of the lack of prior systematic data we did not make formal directional hypotheses. Finally, respondents’ potential solutions to the barriers that they saw as most serious were assessed.

## Materials and methods

### Population and sampling procedures

The purpose of the sampling frame was to identify individuals (A) with moderate to high levels of professional experience in global health, (B) who had considered barriers to global health development, (C) who were focused across a broad range of global health domains, and (D) working in a variety of professional positions (e.g., researcher; upper-level administration at a global health-related organization). We intentionally did not restrict our sample to ‘leaders’ in global health because professionals with extensive ‘on the ground’ experience but without broad international recognition have an important and possibly different perspective. Potential participants were identified via two methods. First, a search was conducted on PubMed for articles published from 2008 to 2013 using the search terms (("global health" OR "low and middle income countries" OR "LMIC") AND (barrier* OR challenge* OR obstacle* OR difficult* OR impediment*) AND development)), restricted to the title and abstract. This produced a total of 408 global health-related articles. Authors of global health-related articles were selected without regard for sub-domain within global health, although authors indicated to not be directly involved in global health itself (e.g., statisticians) were excluded. Second, personnel lists for major global health organizations (e.g., WHO, DFID) were obtained online, and individuals with responsibilities directly involved in global health selected (e.g., accountants were not selected, policy administrators were). The publications and lists were cross-checked within and across methods to avoid duplication. This produced a total of 432 non-duplicated authors for whom it was possible to obtain an active email address (since the survey was to be conducted online and potential participants contacted via email).

Potential participants were contacted with an individualized email describing the study, its purposes and the structure of the study. The email also described the desired study inclusion criteria (e.g., active in global health development), so that individuals not involved directly in global health would not participate. An email reminder was sent two weeks after the initial email to all potential participants. The first page of the online survey involved a consent document describing the survey, its voluntary nature, the anonymity and confidentiality of responses, and whom to contact regarding IRB questions. In order to proceed with the survey participants clicked on an acceptance statement. The study was approved by the Vanderbilt University IRB, with the survey conducted from July 2013 to April 2014.

#### Barriers to global health development survey

Survey questions were extracted from previous articles discussing barriers to global health [2, 3, 6, 7, 11, 12, 13, 14]. Items were then refined with the assistance of several global health experts. Barriers for specific health areas (e.g., cancer [6]) or barriers focused on specific agencies (e.g., the WHO; [7]) were reworded to have a common structure and to focus upon global health broadly. The barriers also were reviewed more generally for wording and comprehensiveness with suggested additional barriers from the experts included after review. The survey was programmed and administered online using the Qualtrics online data collection platform. The survey url was provided in the email sent to potential participants, described above. Individuals were not asked to identify themselves, the specific agency or institution for whom they worked, etc. The survey contains three sets of questions. The first set (*Q1*.*x*) assesses basic demographics as well as global health-related factors. System-level predictors included the (A) *Geographic Region* in which the respondent was working in global health (coded based on World Bank designations [15]), (B) the type of *Home Organization* (e.g., academic institution, NGO) under which the respondent worked, and (C) the *Global Health Domain* on which the participant focused (Table 1 provides specific categories within each of these variables). Individual-level predictors involving participants’ global health-related experience included (D) *Years Working in Global Health*, (E) *Number of Global Health Publications*, (F) *Total Global Health-related Grant Dollars*, (G) in what country the participant’s *Home Office* was located, which was coded as LMIC or HIC based on World Bank designations [15], and (H) the *Number of Weeks In-Country* per year that the participant had spent on average over the past five years. Variables E and F had a Not Applicable response option (e.g., for agency program administrators) which was treated as missing data.

**Table 1:** Respondent characteristics.

Characteristic	Demographic
**Age**	51.3(11.1)
**Female**	111(42%)
**Education level**	
Terminal bachelor’s degree	4(2%)
Terminal master’s degree	50(19%)
PhD degree	123(47%)
MD degree	118(45%)
**Home Organization**	
Academic	138(52%)
Governmental(national) organization	29(11%)
Non-governmental organization (NGO)	39(15%)
Private philanthropic foundation	7(3%)
UN-related	35(13%)
Other	16(6%)
**Years in global health**	14.3(4.9)
**Number global health publications**	34.9(61.2)
**Total grant dollars (U.S.) in global health**	$1,230,973 ($1,168,385)
**Location of Home Office: LMIC (vs. HIC)**	54(21%)
**Mean weeks / year “in-country” over last five years**	20.8(18.9)
**Geographic region of global health development focus**^**1**^	
East Asia and the Pacific	34(13%)
Europe and Central Asia	10(4%)
Latin American and the Caribbean	42(16%)
Middle East and North Africa	7(3%)
South Asia	31(12%)
Sub-Saharan Africa	99(38%)
Global	54(20%)
**Global Health Domain**^**2**^	
Communicable diseases (excluding HIV)	94(36%)
Ethics	43(16%)
Health promotion / public health	73(28%)
HIV	88(33%)
Maternal and child health	113(43%)
Mental health (excluding substance abuse)	41(16%)
NCD (excluding mental health)	58(22%)
Reproductive health	81(31%)
Safety promotion	19(7%)
Substance abuse	33(13%)
Surgery and anesthesia	5(2%)
General global health development	88(33%)

Notes: Data are: mean (SD), or n (%).

^1^ = Based on World Bank global region classification. If two regions were indicated, both were included separately in the table; thus, the sum of the percentages is greater than 100%.

^2^ = Respondents were able to pick more than 1 domain. The mean number of domains per respondent was 2.8 (SD = 2.0), and ranged from 1 to 9.

The second set of questions (*Q2*.*1 to Q2*.*66*) involved ratings (see Table 2) of the extent to which each of the 66 barriers was ‘*Not an important barrier to global health development’* (0) to ‘*A major barrier to global health development’* (4). Study participants were instructed to make the ratings based on their own direct experience within the LMIC(s) within which they worked.

**Table 2:** Barrier rating questions.

**Respondents rate all 66 barriers on:**
**Q2.x**: How much of a barrier is this issue to global health development, in your (the respondent’s) geographic region, for your global health focus (Likert scale 0 to 4)?
**From the highest rated barriers, four most serious for GH development are selected, and rated on:**
**Q3.1**: How difficult will this barrier be to solve (Likert scale 0 to 4)?
**Q3.2:** Impact of barrier on development and / or provision of effective training, research, or health-related services (Likert scale 0 to 4)?
**Q3.3:** Impact of barrier on sustainability of effective programs (Likert scale 0 to 4)?
**Q3.4:** Examples from their own experience of this barrier.
**Q4.5:** Potential solutions for the barrier.

In the third section, the various barriers that the participant rated as a major barrier (‘4’ on the Likert scale) were re-displayed and the participant asked to select from these those they saw as the top 4 barriers. Participants were then asked a final set of questions (*Q3*.*x*), to rate each of these four barriers on a 0–4 scale in regards to (A) how difficult it would be to solve, (B) how much impact the barrier had on development of effective (emphasis included in survey questionnaire) health programs and capacity within the respondent’s global health domain, (C) how much the barrier impacts on sustainability of such programs, (D) examples from their own experience about the barrier, and (E) potential solutions (see Table 2).

### Statistical analysis

Two sets of analyses were conducted. The first involved descriptive statistics (e.g., mean, SD, frequencies) to summarize respondents’ ratings and selections of barriers. The second set of analyses involved inferential statistics to assess relations among various predictors and the barrier seriousness ratings. In these analyses, the *Mean Barrier Rating* refers to the barrier seriousness rating averaged across the 66 barriers (*Q2*.*1* to *Q2*.*66*); this composite score had a Cronbach’s internal consistency alpha of .95. *Barrier Domain* refers to the four barrier factors identified in the exploratory factor analysis (below). To determine whether the levels of these four factors differed as a function of the predictors (e.g., global health focus), repeated measures general linear models (GLM) with *Barrier Domain* as the repeated measure were conducted. The univariate test with the Huynh-Feldt-Lecoutre correction was used for the interaction test. All analyses were conducted using SAS 9.4, including the Proc Factor, Proc Glm, and Proc Means procedures.

## Results

### Response rates, and respondent characteristics

A total of 432 invitations were emailed, 268 participants accessed the website (62% response rate), and 264 (99%) were considered to have complete data for analysis (completed at least 56 of the 66 Q2.x main barrier ratings). Table 1 reports respondent characteristics.

### Exploratory factor analysis on barrier seriousness ratings

**Fig 1:**
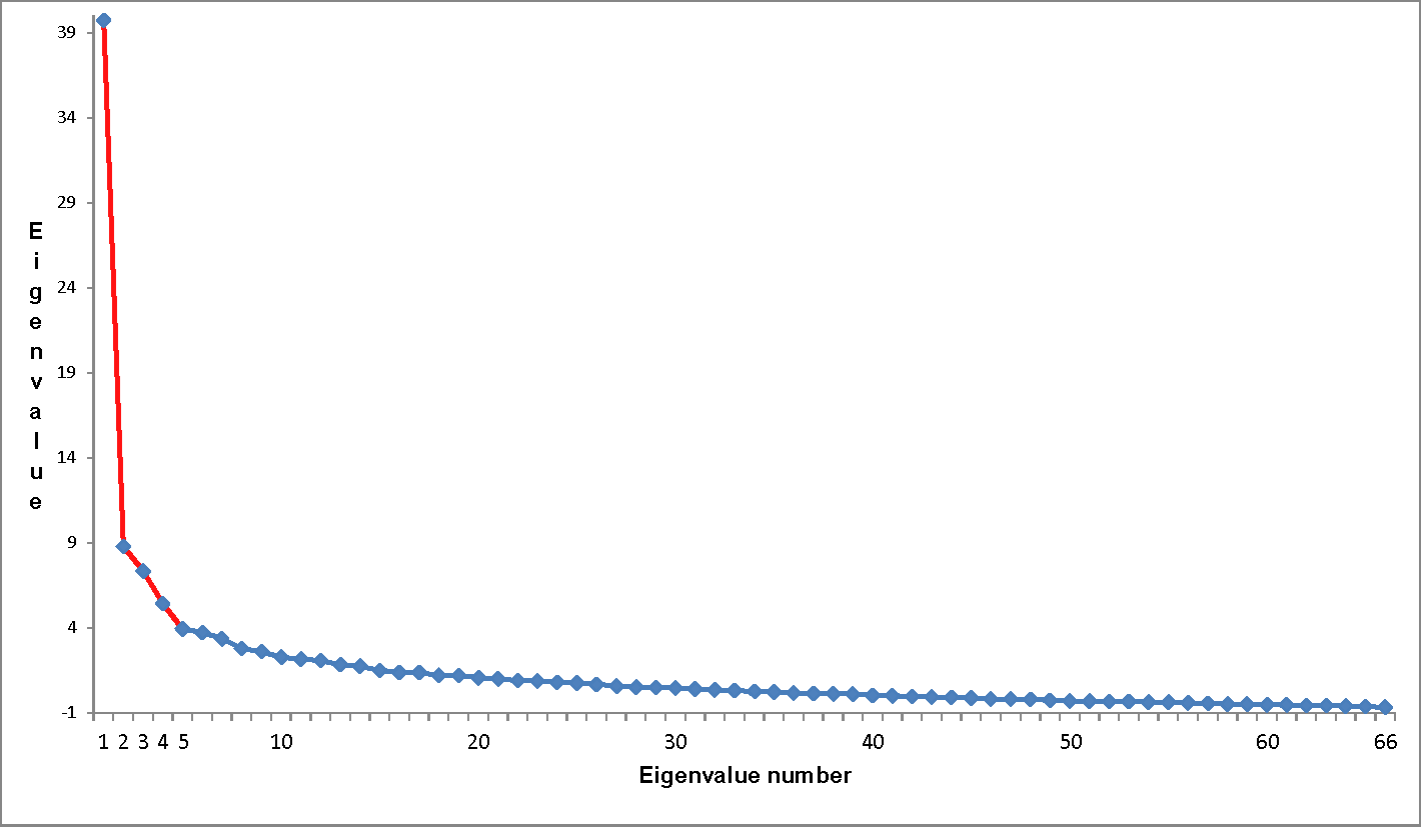
Scree plot from exploratory factor analysis on barrier severity ratings.

We conducted an exploratory factor analysis on the seriousness ratings (Q2.1 –Q2.66) for the 66 barriers. Maximum likelihood factor analysis was used, with each item’s squared multiple correlation with the other items as prior communality estimates, an item loading cutoff criterion of 0.30, and a Promax rotation [16]. The scree plot indicated four factors (see Fig 1, below), which based on the items loading on the factors were labeled: Factor 1, *Corruption*, *Lack of Competence* (e.g., #40. People obtain health care leadership positions through means other than competence and expertise, such as personal connections or corruption) (total variance explained = 20.58; unique variance explained = 9.40); Factor 2, *Priority Selection* (e.g., #26. Non-optimal distribution of funds between prevention, early intervention and tertiary treatment programs) (total variance explained = 15.33; unique variance explained = 5.70); Factor 3, *Resource Limitations* (e.g., #16. Insufficient financial support from domestic sources) (total variance explained = 12.33; unique variance explained = 6.70); Factor 4, *Social and Cultural Barriers* (e.g., #60. People / families are reluctant to seek treatment for this problem because of stigma) (total variance explained = 14.15; unique variance explained = 4.82). The inter-factor correlations are reported in Table 3, and the 66 barriers and the factor loadings are reported in Table 4.

**Table 3:** Inter-factor correlations.

	Factor 1	Factor 2	Factor 3	Factor 4
Factor 1	1.00	0.45	0.33	0.43
Factor 2	0.45	1.00	0.24	0.43
Factor 3	0.33	0.24	1.00	0.31
Factor 4	0.43	0.43	0.31	1.00

**Table 4:** Barriers and factor loadings.

Barrier	Factor 1	Factor 2	Factor 3	Factor 4
43. Health care and development organizations spend funds on things not directly related to the project for which they are funded (e.g., using program money for personal travel; use of funding to support staff unrelated to the project).	0.68	-0.09	0.01	-0.03
31. My global health focus is dominated by western institutions motivated by competitive and financial considerations more than by a true commitment to global health development.	0.64	0.10	-0.08	-0.10
37. Personal self-interest or organizational ambition rather than health care development goals drive organizational and/or programmatic agendas.	0.62	0.04	-0.06	-0.06
46. A lack of openness, honesty and trust as well as a non-collaborative, competitive climate among development partners undermines progress.	0.62	-0.05	-0.15	0.15
48. Foreign experts with relatively little in-country experience believe that they understand the local context and what is needed better than local professionals.	0.62	-0.05	0.11	0.06
41. Funding is provided to NGOs who lack necessary technical expertise for the project; consequently they must hire external technical consultants, resulting in wasted resources as well as project leadership who lack sufficient technical understanding.	0.61	0.03	0.04	0.08
34. Influx of large amounts of foreign funds encourages an ‘under-the-table’ market for local organizations (e.g., local partners expect non-project related incentives in order to participate).	0.61	-0.12	0.27	-0.09
30. Funding is based more on donor goals (e.g., obtaining good publicity for the organization) than on actual LMIC needs.	0.59	0.21	0.04	-0.08
35. Influx of large amounts of foreign funds undermines government stewardship of health care.	0.59	-0.11	0.28	-0.01
47. Agencies or individuals use ideas, technology (e.g., treatment manuals; training materials), etc. without obtaining permission and / or giving appropriate credit or control to its owners, resulting in secretiveness, and a lack of interest in collaborating.	0.57	-0.14	-0.14	0.22
39. Unethical practices in research and development (e.g., data fabrication; manipulating data analysis or project reports to distort results in a desired direction).	0.55	-0.04	-0.02	0.12
51. Some funding agencies make funding decisions on non-scientific bases (e.g., personal relationships; their own personal evaluation rather than expert scientific evaluation).	0.55	0.17	-0.10	-0.03
42. Poor coordination or competition among foreign organizations negatively impacts development (e.g., results in unsustainable, inflated salaries and costs; having to pay patients to participate in treatment programs because of competition to enroll patients.	0.50	-0.01	0.23	0.12
54. Program design and evaluation do not include sufficient consideration of program sustainability post-foreign support.	0.50	0.20	0.07	-0.08
18. Financial resources are provided with excessive external oversight, resulting in inefficiency, project inability to adapt to local realities, and undermining of development of local management autonomy.	0.48	-0.03	0.17	-0.07
36. Influx of large amounts of foreign funds attracts well-trained professionals to programs deemed important by foreign agencies, causing shortages in essential local programs.	0.47	-0.04	0.39	-0.06
40. People obtain health care leadership positions through means other than competence and expertise (e.g., personal connections; corruption).	0.46	0.09	-0.03	0.06
22. Study Tours abroad (trips for LMIC personnel to spend a few weeks overseas), medium-term overseas trainings, etc. end up being little more than paid vacations designed to buy the loyalty of LMIC personnel, wasting development opportunities and scarce resources.	0.45	-0.04	0.09	-0.08
53. Health development organizations excessively focus on program implementation without appropriate pre-project needs assessments, program evaluation, monitoring of outcomes, etc.	0.42	0.32	-0.04	-0.05
45. Organizations or people in global health leadership positions do not understand or acknowledge the significance of certain critical barriers.	0.42	0.24	-0.13	0.09
52. Some health development organizations do not recognize the value of evidence-based methods.	0.41	0.25	-0.11	0.04
50. Your global health focus is seen as not really requiring a high level of formal professional training in order to competently develop, implement, or evaluate programs, resulting in people without appropriate qualifications being active in this area.	0.41	0.24	-0.10	0.10
20. Funding agencies do not fund what is really needed to move the field forward (e.g., because they don`t understand the realities of what is needed, are politically motivated).	0.39	0.36	0.01	-0.08
38. Inadequate professional regulatory / licensing systems results in poor quality control for service provision.	0.39	0.11	0.18	0.02
21. Funding issues (e.g., competition for funding; a lack of stable long-term funding) result in agencies focusing on their survival and on producing quick results, rather than on strategic planning and careful program evaluation.	0.38	0.28	0.20	-0.03
65. Foreign health agency staff do not understand or are not always sensitive to cultural issues when working with local staff.	0.36	-0.03	0.01	0.33
19. Financial resources are provided with inadequate oversight (e.g., poor monitoring of whether funds are spent appropriately; no consequences for mismanagement of funds) and / or financial transparency.	0.35	0.02	0.22	0.09
44. Government health-related policy is inadequate to guide development.	0.31	0.23	-0.09	0.16
7. Agencies and / or individuals attempt to implement health care, research, etc. programs for which they lack the necessary background, training, and / or skills to competently implement the program.	0.30	0.17	0.15	0.03
49. Language barriers.	0.26	0.06	-0.01	0.19
12. Bright, young people are less interested in pursuing careers in this field because of low salaries, low prestige, etc.	0.21	0.15	0.01	0.00
26. Non-optimal distribution of funds between prevention, early intervention and tertiary treatment programs.	-0.14	0.86	0.08	-0.07
27. Non-optimal distribution of funds between centralized, hospital-based programs versus community- based care programs.	-0.15	0.78	0.04	0.08
25. The government prioritizes general economic development over health care development.	0.02	0.58	-0.07	0.00
28. A lack of communication or coordination between different system levels or between different relevant sectors.	0.11	0.43	-0.01	0.18
33. Insufficient involvement of consumers or community beneficiaries in program and research decision making.	0.19	0.40	0.03	0.13
29. Treatment of this health problem is sometimes viewed as not cost effective (e.g., beliefs that treatment of pediatric cancer is not the best use of limited resources).	0.30	0.38	-0.12	0.01
24. Funders and governments focus excessively on specific diseases rather than on development of the health care system.	0.15	0.38	0.20	-0.02
8. The leaders or administrators who make policy and funding decisions do not know the realities of the situation "on the ground".	0.28	0.37	0.01	0.02
23. The professional establishment opposes an expanded role for non-specialists in providing health care.	-0.02	0.36	0.08	0.14
14. Health care and political leaders lack sufficient public health training and experience to appreciate the complexities of health care development in this area.	0.21	0.36	0.09	0.01
15. Insufficient international financial support.	-0.05	0.32	0.22	0.05
32. A focus on easily accessed groups, with less focus on equally or more needy but difficult-to-reach populations.	0.15	0.32	0.10	0.12
17. Too much time must be spent on obtaining funding rather than on program activities.	0.22	0.24	0.06	-0.03
3. Weak physical infrastructure (e.g., poor road construction and physical access; unreliable power supply; weak telecommunication).	-0.06	-0.10	0.80	-0.04
2. Lack of basic life necessities (e.g., clean water, adequate nutrition).	-0.13	0.00	0.77	0.03
4. Weak local health care system / infrastructure may collapse after foreign resources leave.	-0.03	0.06	0.75	0.04
5. Effective medications (including problems related to counterfeit drugs) are unavailable, or are prohibitively expensive.	0.04	0.00	0.64	0.05
1. Violence or political instability.	-0.02	0.24	0.44	0.08
16. Insufficient financial support from domestic sources.	-0.06	0.40	0.39	0.00
10. A lack of trained local specialists (health care workers, researchers, etc.).	0.06	0.06	0.36	0.05
6. Health care professionals provide services for which they lack necessary skills and training, resulting in misdiagnosis, ineffective or iatrogenic treatment, etc.	0.12	0.05	0.36	0.11
11. Professionals go overseas (for training, etc.) and do not return ("brain drain").	0.08	0.02	0.35	0.05
13. Difficulties in getting staff to work and live in rural areas where there is particularly high need.	0.14	0.16	0.23	-0.02
9. A lack of high quality, in-country training programs.	0.18	0.16	0.23	-0.13
61. This health problem or associated behaviors are viewed at least in part as a crime or moral failure, resulting in people being hesitant to seek treatment, and / or the system being ambivalent about providing treatment.	-0.08	0.08	-0.10	0.80
60. People / families are reluctant to seek treatment for this problem because of stigma.	-0.15	0.01	0.02	0.74
56. Incompatibilities between local cultural / religious values and treatment approaches found effective in other countries (e.g., local taboos against discussing sexual issues may be incompatible with STD intervention programs).	-0.02	-0.04	0.27	0.59
62. A lack of protection for basic human rights undermines health development.	0.09	0.19	0.07	0.56
63. Health care workers are reluctant to treat patients for fear of contracting this disease.	0.21	-0.05	-0.01	0.49
66. The media, leaders, etc. misrepresent aspects of this health problem (e.g., the nature, transmission, treatment, etc. of the disease).	0.13	0.14	-0.09	0.46
55. Cultural or religious beliefs about appropriate gender roles interfere with health development (e.g., conflict with global health workers`ability to function to their full professional capacity, or interfere with women`s access to health treatment programs).	-0.06	0.03	0.28	0.46
59. People seek help from local healers, traditional medicine, etc. rather than from scientifically-based treatments.	-0.03	-0.01	0.23	0.45
57. Foreign donors / global health agencies have regulations, values or methods incompatible with local realities (e.g., prohibition of needle exchange programs; a focus on abstinence for prevention of HIV transmission).	0.35	-0.06	0.12	0.40
58. A lack of knowledge among the general public regarding effective treatment options.	0.06	0.23	-0.01	0.29
64. The technical tools used to address my Global Health Focus were created in Western, affluent countries and are less applicable in LMIC.	0.21	0.10	0.00	0.25

### Perceived barrier severity

Table 5 reports the perceived severity of each individual barrier. A repeated measures GLM analysis found that the seriousness ratings (Q2.x) varied significantly (F[65,9620] = 28.31, p<0.0001) across the 66 barriers. A repeated measures GLM analysis also indicated that seriousness ratings varied significantly across *Barrier Domain* (the four factors identified in the exploratory factor analysis) (F[3,789] = 107.96, p<0.0001). Means (and SD) on the 0–4 Likert scale for the four barrier domains, from highest to lowest, were: Factor 3 *Resource Limitations* = 2.47 (0.80); Factor 2 *Priority Selection* = 2.20 (0.75); Factor 1 *Corruption*, *Lack of Competence* = 1.87 (0.74); Factor 4 *Social and Cultural Barriers* = 1.68 (0.84).

**Table 5:** Seriousness ratings for individual barriers.

Mean (SD)		Barrier
3.14	(1.08)	16. Insufficient financial support from domestic sources.
2.79	(1.10)	13. Difficulties in getting staff to work and live in rural areas where there is particularly high need.
2.71	(1.15)	28. A lack of communication or coordination between different system levels or different relevant sectors.
2.68	(1.18)	21. Funding issues (e.g., competition for funding; a lack of stable long-term funding) result in agencies focusing on their survival and on producing quick results, rather than on strategic planning and careful program evaluation.
2.68	(1.30)	4. Weak local health care system / infrastructure may collapse after foreign resources leave.
2.61	(1.23)	54. Program design and evaluation do not include sufficient consideration of program sustainability post-foreign support.
2.57	(1.32)	2. Lack of basic life necessities (e.g., clean water, adequate nutrition).
2.57	(1.25)	24. Funders and governments focus excessively on specific diseases rather than on development of the health care system.
2.54	(1.16)	10. A lack of trained local specialists (health care workers, researchers, etc.).
2.51	(1.23)	3. Weak physical infrastructure (e.g., poor road construction and physical access; unreliable power supply; weak telecommunication).
2.51	(1.09)	9. A lack of high quality, in-country training programs.
2.49	(1.24)	26. Non-optimal distribution of funds between prevention, early intervention and tertiary treatment programs.
2.44	(1.25)	6. Health care professionals provide services for which they lack necessary skills and training, resulting in misdiagnosis, ineffective or iatrogenic treatment, etc.
2.43	(1.24)	8. The leaders or administrators who make policy and funding decisions do not know the realities of the situation "on the ground".
2.41	(1.13)	14. Health care and political leaders lack sufficient public health training and experience to appreciate the complexities of health care development in this area.
2.34	(1.33)	27. Non-optimal distribution of funds between centralized, hospital-based programs versus community- based care programs.
2.29	(1.21)	53. Health development organizations excessively focus on program implementation without appropriate pre-project needs assessments, program evaluation, monitoring of outcomes, etc.
2.29	(1.18)	20. Funding agencies do not fund what is really needed to move the field forward (e.g., because they don`t understand the realities of what is needed, are politically motivated).
2.20	(1.17)	33. Insufficient involvement of consumers or community beneficiaries in program and research decision making.
2.19	(1.22)	40. People obtain health care leadership positions through means other than competence and expertise (e.g., personal connections; corruption).
2.17	(1.16)	7. Agencies and / or individuals attempt to implement health care, research, etc. programs for which they lack the necessary background, training, and / or skills to competently implement the program.
2.17	(1.30)	44. Government health-related policy is inadequate to guide development.
2.16	(1.34)	25. The government prioritizes general economic development over health care development.
2.14	(1.42)	1. Violence or political instability.
2.14	(1.18)	11. Professionals go overseas (for training, etc.) and do not return ("brain drain").
2.14	(1.21)	15. Insufficient international financial support.
2.12	(1.17)	17. Too much time must be spent on obtaining funding rather than on program activities.
2.10	(1.28)	48. Foreign experts with relatively little in-country experience believe that they understand the local context and what is needed better than local professionals.
2.09	(1.18)	5. Effective medications (including problems related to counterfeit drugs) are unavailable, or are prohibitively expensive.
2.06	(1.22)	46. A lack of openness, honesty and trust as well as a non-collaborative, competitive climate among development partners undermines progress.
2.04	(1.28)	45. Organizations or people in global health leadership positions do not understand or acknowledge the significance of certain critical barriers.
2.02	(1.27)	12. Bright, young people are less interested in pursuing careers in this field because of low salaries, low prestige, etc.
2.02	(1.38)	62. A lack of protection for basic human rights undermines health development.
2.00	(1.33)	60. People / families are reluctant to seek treatment for this problem because of stigma.
1.99	(1.33)	18. Financial resources are provided with excessive external oversight, resulting in inefficiency, project inability to adapt to local realities, and undermining of development of local management autonomy.
1.98	(1.21)	58. A lack of knowledge among the general public regarding effective treatment options.
1.96	(1.37)	30. Funding is based more on donor goals (e.g., obtaining good publicity for the organization) than on actual LMIC needs.
1.92	(1.33)	36. Influx of large amounts of foreign funds attracts well-trained professionals to programs deemed important by foreign agencies, causing shortages in essential local programs.
1.91	(1.30)	38. Inadequate professional regulatory / licensing systems results in poor quality control for service provision.
1.90	(1.26)	55. Cultural or religious beliefs about appropriate gender roles interfere with health development (e.g., conflict with global health workers`ability to function to their full professional capacity, or interfere with women`s access to health treatment programs).
1.90	(1.30)	19. Financial resources are provided with inadequate oversight (e.g., poor monitoring of whether funds are spent appropriately; no consequences for mismanagement of funds) and / or financial transparency.
1.89	(1.36)	42. Poor coordination or competition among foreign organizations negatively impacts development (e.g., results in unsustainable, inflated salaries and costs; having to pay patients to participate in treatment programs because of competition).
1.86	(1.25)	37. Personal self-interest or organizational ambition rather than health care development goals drive organizational and/or programmatic agendas.
1.85	(1.27)	32. A focus on easily accessed groups, with less focus on equally or more needy but difficult-to-reach populations.
1.84	(1.15)	56. Incompatibilities between local cultural / religious values and treatment approaches found effective in other countries (e.g., local taboos against discussing sexual issues may be incompatible with STD intervention programs).
1.82	(1.27)	52. Some health development organizations do not recognize the value of evidence-based methods.
1.75	(1.37)	35. Influx of large amounts of foreign funds undermines government stewardship of health care.
1.75	(1.24)	51. Some funding agencies make funding decisions on non-scientific bases (e.g., personal relationships; their own personal evaluation rather than expert scientific evaluation).
1.74	(1.23)	41. Funding is provided to NGOs who lack necessary technical expertise for the project; consequently they must hire external technical consultants, resulting in wasted resources as well as project leadership who lack sufficient technical understanding.
1.69	(1.25)	57. Foreign donors / global health agencies have regulations, values or methods incompatible with local realities (e.g., prohibition of needle exchange programs; a focus on abstinence for prevention of HIV transmission).
1.69	(1.30)	34. Influx of large amounts of foreign funds encourages an ‘under-the-table’ market for local organizations (e.g., local partners expect non-project related incentives in order to participate).
1.65	(1.16)	59. People seek help from local healers, traditional medicine, etc. rather than from scientifically-based treatments.
1.64	(1.42)	61. This health problem or associated behaviors are viewed at least in part as a crime or moral failure, resulting in people being hesitant to seek treatment, and / or the system being ambivalent about providing treatment.
1.60	(1.27)	29. Treatment of this health problem is sometimes viewed as not cost effective (e.g., beliefs that treatment of pediatric cancer is not the best use of limited resources).
1.57	(1.18)	65. Foreign health agency staff do not understand or are not always sensitive to cultural issues when working with local staff.
1.57	(1.28)	49. Language barriers.
1.55	(1.16)	66. The media, leaders, etc. misrepresent aspects of this health problem (e.g., the nature, transmission, treatment, etc. of the disease).
1.54	(1.26)	23. The professional establishment opposes an expanded role for non-specialists in providing health care.
1.49	(1.31)	50. Your global health focus is seen as not really requiring a high level of formal professional training in order to competently develop, implement, or evaluate programs, resulting in people without appropriate qualifications being active in this area.
1.47	(1.37)	31. My global health focus is dominated by western institutions motivated by competitive and financial considerations more than by a true commitment to global health development.
1.37	(1.18)	22. Study Tours abroad (trips for LMIC personnel to spend a few weeks overseas), medium-term overseas trainings, etc. end up being little more than paid vacations designed to buy the loyalty of LMIC personnel, wasting development opportunities and scarce resources.
1.25	(1.17)	43. Health care and development organizations spend funds on things not directly related to the project for which they are funded (e.g., using program money for personal travel; use of funding to support staff unrelated to the project).
1.23	(1.17)	64. The technical tools used to address my global health focus were created in Western, affluent countries and are less applicable in LMIC.
1.21	(1.20)	39. Unethical practices in research and development (e.g., data fabrication; manipulating data analysis or project reports to distort results in a desired direction).
1.10	(1.09)	47. Agencies or individuals use ideas, technology (e.g., treatment manuals; training materials), etc. without obtaining permission and / or giving appropriate credit or control to its owners, resulting in secretiveness, and a lack of interest in collaborating,
0.74	(0.97)	63. Health care workers are reluctant to treat patients for fear of contracting this disease.

### Global health development model

In Weiss et al.’s [9] sustainable global health development model, the twin *Capacity Development Goals* of (A) Development of Effective Programs, and (B) Program Sustainability are linked to long-term development success. In the next analyses, we assessed the extent to which these core factors (program efficacy, Q3.2; program sustainability, Q3.3) as well as the difficulty the barrier presented to be solved (Q3.1) were related to perceptions of barrier seriousness. The 66 barriers served as the observations, with each barrier being assigned its mean seriousness rating (Q2.x) across the 264 respondents, as well as the mean across respondents for the Q3.1 to Q3.3 ratings (above). We conducted two analyses. In the first, a series of three simple (1 predictor variable) weighted regression analyses were conducted, with the dependent variable the barrier seriousness rating (Q2.x), and the predictor variables Q3.1, Q3.2, Q3.3. In this as well as the second analysis (below), because different barriers had different numbers of respondents (i.e., were selected by different number of respondents for the Top 4), weighted least squares analysis was used, with the weight equal to the square root of the sample size [17] for the item (i.e., the number of participants who had rated the item for Q3.1, Q3.2, Q3.3, having selected it in the Top 4).

Results for the three simple regressions with standardized beta were: Q3.1 β(Difficulty Solving Barrier) = .07 (F[1,62] = 0.29, ns); Q3.2 β(Impact on Development of Effective Programs) = .10 (F[1,62] = 0.57, ns); Q3.3 β(Impact on Sustainability) = .44 (F[1,62] = 14.76, p< .0003). In the second analysis, to assess unique (as opposed to total) effects, a weighted multiple regression was used. The overall model was significant (F[3,60] = 5.06, p<0.004) with a moderate effect size (R^2^ = 0.20). Again, Impact on Sustainability was significant (β = 0.48, F[1,60] = 14.44, p<0.0003) but the other two predictors were not, β(Difficulty Solving Barrier) = -0.08 (F[1,60] = 0.37, ns), β(Impact on Development of Effective Programs) = -0.05 (F[1,60] = 0.13, ns). The three β differed significantly (F[2,124] = 4.77, p<0.02). Overall, then, it appears that perceptions of barriers’ seriousness are related to the sustainability but not to the development of effective programs or difficulty solving the barrier.

### Predictors of perceived barrier severity

In these analyses, we investigated four potential predictors of perceived barrier seriousness: the respondents’ (1) Organization Type, (2) Geographic Region of global health work, (3) Global Health Focus, and (4) Global Health Experience. Two analyses were conducted for each predictor. The first focused on main effects, and assessed the extent to which overall perceived barrier seriousness (the *Mean Barrier Rating*, the mean of the 66 barrier seriousness ratings, Q2.1 to Q2.66) differed as a function of the predictor; e.g., it assessed the extent to which respondents with, for instance, different global health focus differed in their perceptions of global health barriers’ overall seriousness. The second analysis assessed the extent to which effects of the predictor variable (e.g., the respondent’s global health focus) on perceived barrier seriousness differed as a function of *Barrier Domain*. This analysis used a repeated measure GLM, including the main effect of the predictor variable, *Barrier Domain* (i.e., the 4 barrier factors identified above in the exploratory factor analysis), and their interaction.

#### Organization type

In the analysis of Organization Type (for which the respondent worked; i.e., academic institution; government organization; NGO; philanthropic organization; UN-related organization), individuals selecting ‘Other’ (approximately 8%) were not included in the analysis. The effect of Organization Type on the overall perceived barrier seriousness (the *Mean Barrier Rating*) was non-significant (F[4,243] = 2.31, ns). The interaction between the respondents’ type of organization and *Barrier Domain* also was non-significant (F[12,729] = 1.24, ns). Thus, respondents from different types of organizations did not differ significantly in the relative seriousness with which they perceived the global health barriers, either overall or as a function of the four different barrier domains.

#### Geographic region

This analysis assessed whether the perceived seriousness of barriers differed as a function of the World Bank-defined (above) regions. Respondents reporting a ‘global’ geographic focus (as opposed to a specific region) and respondents reporting more than one World Bank regional focus (n = 59, 23%) were not included in these analyses since these respondents’ perceptions would be influenced by multiple regions and hence difficult to interpret. The effect of Region on the *Mean Barrier Rating* was non-significant (F[5,193] = 1.32, ns). However, the interaction between *Barrier Domain* and Region was significant (F[15,579] = 4.82, p<0.0001), indicating that professionals working in the different Regions viewed the four barrier domains as having significantly different seriousness. Univariate analyses indicated that the effect of Region was significant for *Resource Limitations* (Factor 3; F[5,193] = 6.25, p<0.0001), and *Social and Cultural Barriers* (Factor 4; F[5,193] = 3.48, p<0.005) but not for the other 2 barrier domains. *Sub-Saharan Africa* had the highest level of perceived seriousness for *Resource Limitations*, significantly higher than *East Asia and Pacific*, *South Asia*, *Latin America and the Caribbean* (see Table 6). The *Middle East and North Africa* region had the highest level of perceived seriousness for the *Social and Cultural Barriers*, significantly higher than for *Latin America and the Caribbean; Sub-Saharan Africa* had the second highest level on this factor and also was significantly higher than *Latin America and the Caribbean*.

**Table 6:** Mean and SD for barrier factor ratings with significant region effects.

Region	n	Factor 3		Factor 4	
*East Asia and the Pacific*	29	2.00^a^	(0.95)	1.56^ab^	(0.75)
*Europe and Central Asia*	6	2.11^ab^	(0.94)	1.68^ab^	(0.47)
*Latin America and the Caribbean*	41	1.97^a^	(0.77)	1.18^b^	(0.85)
*Middle East and North Africa*	5	2.31^ab^	(0.40)	2.36^a^	(1.04)
*South Asia*	27	2.11^a^	(0.99)	1.69^ab^	(0.81)
*Sub-Saharan Africa*	91	2.64^b^	(0.63)	1.72^a^	(0.84)

Notes: Factor 3 = *Resource Limitations*. Factor 4 = *Social and Cultural Barriers*. Respondents with a “global” regional focus, including those selecting more than 1 region, were not included in this analysis. Within columns (Factor), regions with the same subscript do not differ significantly for this factor.

#### Global health domain

To determine whether overall perceived barrier serious (*Mean Barrier Rating*) differed as a function global health domain, a series of 12 one-way ANOVA analyses (across the 12 global health domains) were conducted. This approach, rather than treating Global Health Domain as a single 12 category factor, was taken because many respondents reported being active in more than 1 global health domain (mean number of domains = 2.8, SD = 2.0, range = 1 to 9), creating statistical non-independence for a single Global Health Domain factor. Of the 12 domains, only General Global Health Development showed a significant effect, with respondents involved in this domain reporting significantly (F[1,262] = 4.54, p<0.05) higher overall barrier seriousness than those respondents not involved in this domain (2.12 vs. 1.95, respectively, on the 0 to 4 Likert scale).

We next assessed extent to which perceptions of the relative seriousness of the four Barrier Domains differed as a function of the whether or not the respondent was active in a particular global health domain. Of the 12 interaction tests, 5 were significant (see Table 7). For Substance Abuse, the interaction reflected the fact that only Factor 3 showed a significant effect, with individuals involved in the Substance Abuse domain reporting lower levels of *Resource Limitations* barriers than individuals not involved in the Substance Abuse domain. For HIV, the interaction reflected the fact that only Factor 4 showed a significant effect, with individuals involved in the HIV domain reporting higher levels of *Social and Cultural Barriers* than individuals not involved in the HIV domain. Mental Health showed a similar pattern, with individuals involved in Mental Health reporting higher levels of *Social and Cultural Barriers* than individuals not involved in Mental Health, and effects on the 3 other factors non-significant. For the interactions involving the Communicable Diseases domain and the Non-Communicable Diseases domain, none of the individual univariate tests were significant. The significant interactions reflected the fact that the trends across the Barrier Domains ran in opposite directions. For the Communicable Diseases domain, *Priority Selection* barriers (Factor 2) were (non-significantly) lower for those involved in this domain than those not involved in this domain (see Table 7) whereas *Resource Limitations* barriers (Factor 3) were seen as (non-significantly) higher by those involved in this domain. For the Non-Communicable Diseases domain, *Resource Limitations* barriers (Factor 3) were lower for those involved in this domain than those not involved in this domain (see Table 7) whereas *Social and Cultural Barriers* (Factor 4) were seen as higher by those involved in this domain.

**Table 7:** Significant barrier domain x health domain interactions.

Global Health Domain: F test^1^	Group	Factor 1 Mean (SD)	Factor 2 Mean (SD)	Factor 3 Mean (SD)	Factor 4 Mean (SD)
Substance Abuse: F(3,786) = 3.36*	0	1.89(0.74)	2.22(0.74)	2.52(0.77) **	1.68(0.86)
	1	1.79(0.72)	2.05(0.82)	**2.13(0.92)**	1.74(0.71)
Communicable Diseases: F(3,786) = 2.95*	0	1.86(0.76)	2.25(0.75)	2.43(0.79)	1.69(0.86)
	1	1.91(0.69)	2.11(0.75)	2.56(0.80)	1.67(0.81)
HIV: F(3,786) = 4.21**	0	1.86(0.76)	2.23(0.74)	2.43(0.82)	1.59(0.86) *
	1	1.90(0.71)	2.15(0.78)	2.55(0.74)	**1.86(0.77)**
Mental Health: F(3,786) = 2.95*	0	1.85(0.71)	2.18(0.73)	2.48(0.81)	1.63(0.80) *
	1	2.00(0.87)	2.33(0.84)	2.43(0.73)	**1.97(0.98)**
NCD (excluding mental health):F(3,786) = 2.92*	0	1.85(0.75)	2.19(0.76)	2.51(0.79)	1.72(0.85)
	1	1.94(0.70)	2.26(0.72)	2.34(0.82)	1.57(0.79)

Notes: Ethics, Public Health Promotion, Maternal and Child Health, Reproductive Health, Safety Promotion, Surgery and Anesthesia, and General Global Health did not have significant interactions with *Barrier Domain*. Highlighted cells show significant effects for Group = 0 vs. Group = 1 comparison for this Global Health Domain, and this barrier factor

* = p < .05

** = p < .005.

^1^ **=** F test for Global Health Domain X Barrier Domain interaction. Group = 1 indicates respondent reported working in this Global Health Domain, Group = 0 indicates not working in this Global Health Domain. Factor 1 = *Corruption*, *Lack of Competence*. Factor 2 = *Priority Selection*. Factor 3 = *Resource Limitations*. Factor 4 = *Social and Cultural Barriers*.

#### Global health experience

**Fig 2:**
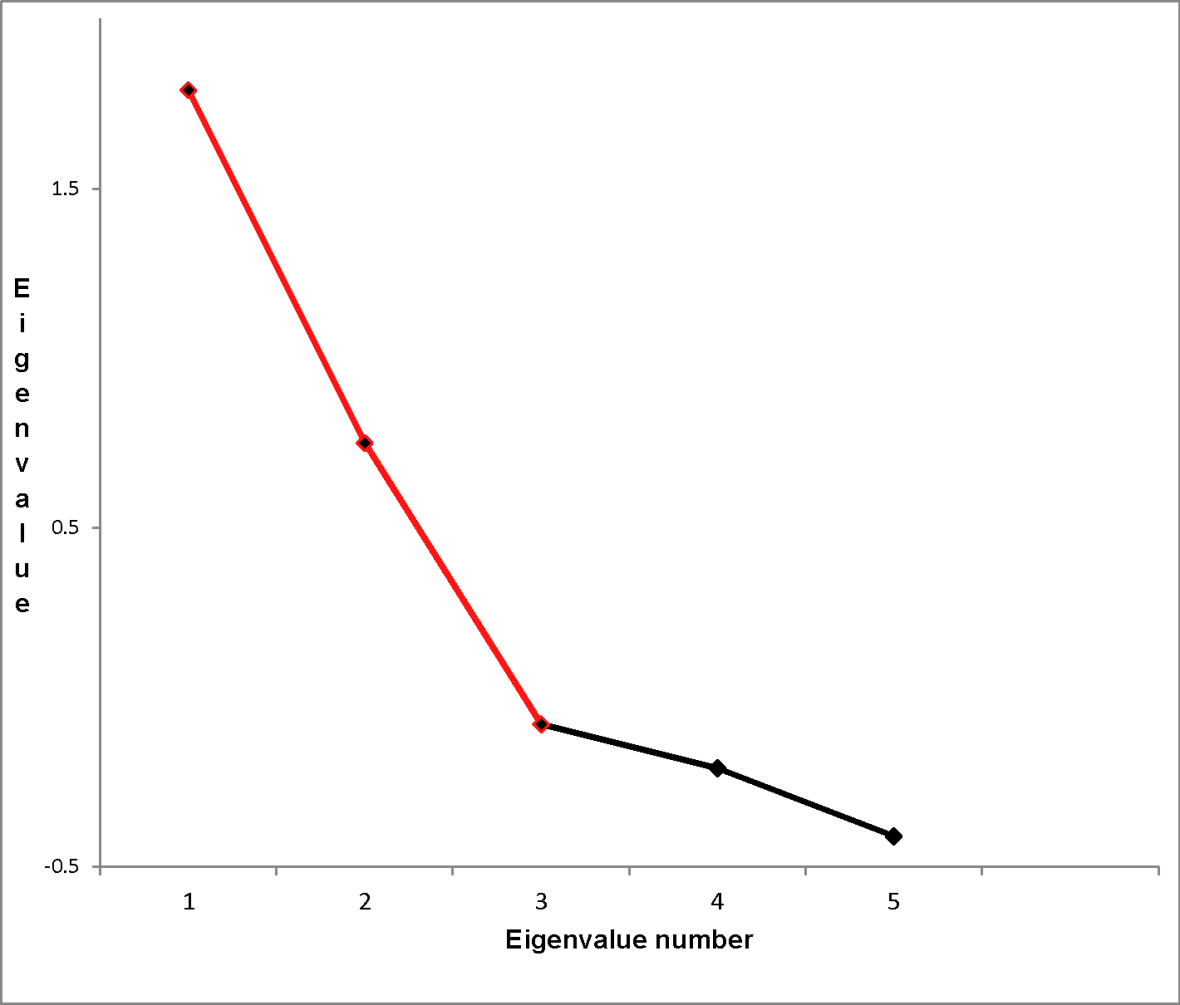
Scree plot for exploratory factor analysis on global health experience variables.

The final analyses assessed the extent to which perceived barrier seriousness varied as a function of the five global health experience variables (Years in Global Health; Number of Publications in Global Health; Total Grant Dollars in Global Health; whether the participant’s Home Office was located in an LMIC or HIC, dummy coded as LMIC = 1, HIC = -1 for the various analyses below; mean weeks spent “In-Country” per year over the past five years). In order to identify underlying dimensions, we conducted an exploratory factor analysis on these variables using maximum likelihood factor analysis, with each of the five variable’s squared multiple correlation with the other variables as prior communality estimates, an item loading cutoff criterion of 0.30, and a Promax rotation [16]. The scree plot indicated two factors (see Fig 2 below), which based on the item loadings (see Table 8) were labeled: Factor 1, *LMIC-oriented* (variables loading on this factor were *Weeks In-Country*, and *Home Office*) (total variance explained = 1.43; unique variance explained = 1.32); Factor 2, *Academic Global Health Achievement* (variables loading on this factor were *Number of GH Publications*, *Total GH Grant Dollars*, *Years in Global Health*) (total variance explained = 1.04; unique variance explained = 0.99). The two factors were negatively correlated with each other, -.20.

**Table 8:** Factor loadings for global health experience variables.

Variable	Factor1	Factor2
Weeks In Country	1.00	0.03
Home Office in LMIC (or HIC)	0.59	-0.05
Number of Global Health Publications	-0.09	0.62
Total Global Health Grant Dollars	0.08	0.59
Years in Global Health	-0.04	0.41

The first analysis with these two factors assessed their total effects in relation to the *Mean Barrier Rating*, in simple (1 predictor) regression equations.

Both tests were significant, with β(*LMIC-oriented*) = 0.12, (F[1,262] = 3.90, p<0.02) and β(*Academic Global Health Achievement*) = -.14, F(1,262] = 5.09, p < .03). Unique effects were assessed in a multiple regression equation including both predictors. The overall model was significant (F[2,261] = 4.15, p < .02, R^2^ = .03), and the individual parameter estimate for *Academic Global Health Achievement* was significant, (F[1,261] = 4.37, p < .04, β = -.13). In the repeated measures GLM, the interaction between *Barrier Domain* and *LMIC-oriented* was significant, (F[3,786] = 5.32, p < .002, R^2^ = .01). Underlying this interaction was the fact that the effect of *LMIC-Oriented* was significant (a) for Factor 1 (*Corruption*, *Lack of Competence*), with β(*LMIC-oriented*) = 0.17, F[1,262] = 7.55, p<0.007, and (b) for Factor 2 (*Priority Selection*), with β(*LMIC-oriented*) = 0.13, F[1,262] = 4.71, p<0.04, but the effect for *LMIC-oriented* was (c) not significant for Factor 3 (*Resource Limitations*) and Factor 4 (*Social and Cultural Barriers*). The interaction between *Barrier Domain* and *Academic Global Health Achievement* was not significant, indicating that the relation between *Academic Global Health Achievement* and perceived barrier severity did not differ significantly as a function of *Barrier Domain*. In sum, these analyses suggest that global health professionals who spend more time in-country and whose Home Office is in an LMIC tend to view (a) corruption and lack of competence, and (b) priority selection as more significant barriers in comparison to global health professionals without these characteristics. However, the effect of Academic Professional Global Health Achievement ran in the opposite direction, with participants with higher levels of academic global health achievement (e.g., more GH publications) seeing lower barrier seriousness than professionals with lower levels of experience in this area, and this effect did not differ significantly across barrier domain.

### Potential solutions

The final question that respondents answered for their Top 4 barriers asked for potential solutions to the barriers the respondent had selected. Solutions for the five barriers most frequently selected as being in the Top 4 were reviewed, similar solutions combined, and summarized in Table 9. One relatively minor but important theme (not included in Table 9 because it does not represent a ‘solution’) mentioned by several respondents in regards to these barriers was that although they likely are most obvious and present the greatest difficulties in LMIC, the barriers also occur in HIC. One respondent stated, for instance, ‘I do not believe these issues are unique to the situation where I work. Similar issues may be raised for more developed economies, including that of the United States.’ A second relatively minor but important theme involved several respondents reflecting on the seemingly overwhelming complexity of some of the barriers, with one participant stating for instance ‘I have no idea [how this barrier might be addressed]. I feel so beaten down by this I think one just has to keep working around these barriers and doing one thing at a time that may have an impact on health.’ Similarly, other respondents noted that the political complexity of many of these barriers seemingly placed them beyond the direct influence of global health development, stating for instance ‘This barrier [weak physical infrastructure] is a geopolitical issue that is beyond the ability of health care deliverers, researchers and policy makers to address. We can only collectively continue to assert the obvious importance of health to a nation's development.

**Table 9:** Proposed solutions for the five most frequently selected Top 4 barriers.

**Top barrier #1 (Barrier 16. *Insufficient financial support from domestic sources*)** • Require increased domestic government funding through matching funding requirements (even if at a very low level) for specific projects, and / or international agencies (e.g., IMF, WB) should mandate levels of GDP that countries should use for their health systems in order to receive IMF, etc. funds. • Train the health sector in financial negotiation skills so that it can obtain increased government funding. • Using empirical health data, increase awareness in local governments of the general economic benefits for the country of health care investment. • Focus on health topics / domains in which the country itself already is and is willing to invest, rather than in what foreign agencies are most interested. • Enhance the health advocacy role of media, NGO, etc.
**Top barrier #2 (Barrier 4. *Weak local health care system / infrastructure may collapse after foreign resources leave*)** • Funding and development agencies should focus on health care system strengthening rather than on specific diseases or disease domains (i.e., focus on ‘horizontal’ rather than ‘vertical’ development). • Global health professionals and agencies should take a long-term view (i.e., 20 years rather than 5 years) for development and investment. • Demonstrate the value of health care in financial as well as human terms, not only to the health policy makers but also to financial and political leaders in the government. • Collaborate with local and national governments to identify their priorities, and focus on these so that development efforts map onto the country’s long-term goals. • Support public-private partnerships that link corporate interests to public health development.
**Top barrier #3 (Barrier 2. *Lack of basic life necessities; e*.*g*., *clean water*, *adequate nutrition)*** • Provide education to local health workers and communities that will increase the efficiency (vis-a-vis use of basic life necessities) of nutrition, sanitation, prenatal care, etc. practices that are related to health. • Develop cross-sector collaboration in health development, working with agencies directly responsible for clean water, etc. • Engage local leaders in management of essential commodities. • Health development activities should target fundamental social determinants of health and disease, including education, poverty and gender equity, which ultimately will increase availability of basic life necessities.
**Top barrier #4 (Barrier 3. *Weak physical infrastructure; e*.*g*., *poor road construction and physical access; unreliable power supply; weak telecommunication*)** • Work to ensure that linkages between (a) infrastructure development undertaken by international agencies (e.g., the WB), and (b) potential health impacts of the infrastructure development are taken into consideration when making planning decisions regarding the infrastructure development (e.g., when evaluating whether to build a new road, increased access to medical services might be one potential benefit of the road considered). • Adopt a cross-sector approach to health development, from the local level to the highest national policy levels, that increases awareness of the relation between health, social determinants of health, and physical infrastructure. • Emphasize the importance of health for general economic development, and in turn the effects of physical infrastructure on health.
**Top barrier #5 (Barrier 1. *Violence or political instability*)** • Support development of a technocratic, merit-based system and government, in and through one’s own health development work. Work to promote health leaders and senior officers based on merit and expertise, rather than on political power, etc. • Diversify one’s health collaborators, institutions, geographic sites within the country / region, etc. in order to reduce potential effects of instability and violence to one’s health development program. Include relatively stable areas to support program durability and survival but also more high-risk areas to maintain critical connections in politically important but unstable areas. • Focus on local-level activities to offset instability at the national political level. Focus health development activities and funding towards academic institutions rather than governments as universities may more stable and have a more robust accountability structure. • Support education generally, particularly for girls who are most likely to use education to benefit their local population, and increase its stability and resiliency. • The health sector cannot really address this directly, but we should be aware of these potential events when planning our strategies.

## Discussion

To the best of our knowledge, this is the first broad-based, quantitative, international survey of barriers to general global health development, assessing correlates of global health professionals’ perceptions of barriers to global health development. Thirty-four of 66 barriers were seen as moderately serious (the midpoint on the scale) or above, highlighting the widespread, significant challenges that global health development faces. Our factor analysis indicated four factors underlying the ratings of the 66 barriers’ seriousness, which in order of the seriousness of the barrier as reported by our respondents were: (A) *Resource Limitations*, (B) *Priority Selection*, (C) *Corruption*, *Lack of Competence*, and (D) *Social and Cultural Barriers*. As factor analysis identifies clusters of items that tend to covary [16] these factors may be useful for researchers and other global health professionals by providing easier and more efficient identification and response to sets of barriers rather than focusing on, at least initially, a larger number of individual barriers. One area for future research suggested by these results might be analysis of directional relations among the different barrier domains. For instance, one might anticipate that higher levels of corruption-related barriers in a particular LMIC could result in higher levels of resource barriers in that same LMIC, as scarce resources were wasted due to corruption. However, conversely, it is quite possible that resource limitations in turn can increase risk for corruption, as individuals in positions of power (e.g., policy-makers; health-care providers) abuse their power to supplement their income through bribery, extortion, etc. This of course can occur not just in LMIC but also in HIC when one puts self-interest above public responsibility but may be more like to occur in LMIC where many individuals, even health-care providers and policy-makers, struggle to make a decent living [18]. Such processes could be particularly harmful as they create self-sustaining feedback loops; i.e., as more resources are wasted and unavailable due to corruption, there likely will be even more pressure on individuals in positions of power to put self-interest above public responsibility, in turn wasting more resources, etc. Similarly, higher levels of social and cultural barriers in a particular LMIC could in turn lead to higher priority selection barriers as the cultural issues influenced selection of health-care priorities, beyond evidence-based priority selection. Priority selection barriers fundamentally represent differences of opinion regarding what should be prioritized, and differences in cultural values (e.g., between the host country and foreign partners) could lead to differences of opinion regarding upon which health care targets, programs, etc. one should focus. Understanding such causal sequencing of barrier domains would be a complex research task since these are society-level variables and factors that would be difficult to manipulate, but understanding this causal sequencing of the barriers would be a very useful component in addressing them.

Contrary to expectations, in our study most system-level predictors showed non-significant or relatively limited relations to barrier seriousness. Perceptions of barrier seriousness did not vary across the type of organization for which the respondent worked. This is positive, in that it suggests that general perceptions of barriers are not an issue potentially resulting in some level of lack of coordination and reduced efficiency across different types of agencies in the field. It also suggests that, although professionals in different agencies may sometimes view the perspective of their agency as ‘most accurate’, in general perceptions of individuals in different types of agencies are not substantially different, at least in regards to global health barriers.

We found a relatively small number of significant differences as a function of Geographic Region. There were no significant regional differences overall, or in regards to *Corruption*, *Lack of Competence* (Factor 1) and *Priority Selection* (Factor 2) barriers. It is not surprising that Sub-Saharan Africa showed the highest levels of higher *Resource Limitations* (Factor 3), given the general high levels of poverty in this region. *Middle East and North Africa*, and *Sub-Saharan Africa* showed the highest levels of *Social and Cultural Barriers* and were significantly higher than Latin America and the Caribbean.

These geographic region effects may have had an influence on the relative levels of the different barrier domains in the overall sample (i.e., collapsed across region). Likely reflecting at least in part the distribution of global health activity, over a third of our sample (38%) reported working in Sub-Saharan Africa. This in conjunction with the fact that Sub-Saharan Africa reported the highest seriousness for Resource Limitations barriers may one reason why Resource Limitations were the barrier domain with the highest average seriousness rating in the overall sample. Conversely, also likely at least in part reflecting the distribution of global health work, only 3% of our sample reported focusing on the Middle East and North Africa region. This in conjunction with the fact that the Middle East and North Africa was the region that reported the highest levels of Social and Cultural Barriers may in part underlie why Social and Cultural Barriers was the barrier domain with the lowest average seriousness rating in the overall sample. This highlights the importance of considering such interaction effects (e.g., Barrier Domain X Geographic Region) when interpreting results.

Several effects for global health domain were significant. Given the high levels of stigma associated with HIV [19], it is not surprising that professionals working in this area reported higher levels of cultural and social barriers. Professionals working in Mental Health similarly reported higher levels of cultural and social barriers, also likely reflecting domain-specific stigma, and highlighting some of the challenges particularly important for these two domains. Individuals working in Substance Abuse reported fewer *Resource Limitations* barriers, perhaps because only 19% of respondents working in the substance abuse area (vs. 38% in the overall sample) were working in Sub-Saharan Africa, which had the highest level of resource limitation barriers.

Perceived barrier seriousness was significantly related to the impact of the barrier on program sustainability but not to barrier impact on development of effective programs, or to difficulty solving the barrier. One possible explanation for the former finding is that among our sample of relatively experienced GH professionals, effective programs have been developed and are at least initially in place, and key barriers now are related to program sustainability. This also may reflect the increasing emphasis and awareness of the importance of sustainability in the literature [20]. In any case, it is positive that sustainability is a central concern linked to GH barrier seriousness.

Global health experience showed complex but interesting relations to perceptions of barrier seriousness. The higher the level of being *LMIC-oriented* (i.e., spending more time in-country, having a Home Office in an LMIC) that participants reported, the higher the levels of *Corruption*, *Lack of Competence* barriers and *Priority Selection* barriers they reported. In contrast, the higher the levels of *Academic Global Health Achievement* (e.g., more GH publications; more GH grant dollars) that participants reported, the lower the level of barrier seriousness that participants reported across all barriers (i.e., the effect did not differ significantly across barrier domain). The *LMIC-oriented* effect might reflect the fact that whether slowness or lack of success in a development target is due a barrier, or simply to the complexity of the development task, is not always immediately clear. For instance, if progress is relatively slow in gaining official approval for a new program, it may not immediately be apparent that this is due to the inherent complexity of the task (needing significant time to carefully review a proposed new program in order to fully understand its potential impact on the population, etc.), or due to corruption or incompetence, or a priority problem. One might expect that global health professionals more closely connected to the LMIC might be more aware of such factors underlying this lack of success. On the other hand, global health professionals who are more successful academically may be, by definition of our variable, more focused on research publications and grants with their perceptions of their academic success generalizing to the situations in the LMIC (i.e., the more successful they have been able to be academically, the fewer barriers they perceive due to generalization of their success or perceptions of success), or reflecting their actual ability to be successful. Regardless of their interpretation, these contrasting effects highlight the importance of having a broad global health team, with both with members with close LMIC connections as well as members capable of academic success (which of course may reside within the same person, although these two variables were negatively correlated in our data, albeit at a low level, -.20).

One of the most important parts of the survey was respondents’ suggestions for potential solutions for the top barriers (see Table 9). Our purpose in this was not to identify solutions that were necessarily ‘innovative’ for the field, but rather to provide a broad range of potential solutions that our respondents saw as at least potentially useful and that other GH professionals could review, and consider applying in their own settings as they saw fit. One area for future development activity might be to develop brief, online toolkits around these solutions (e.g., a one page outline of how this solution has been applied by other GH professionals) with open online access, linked to a brief survey evaluating how the toolkit had been applied, and the perceived utility of the toolkit. Such toolkits could be useful to help us all avoid ‘reinventing the wheel’ unnecessarily. The secondary comments in the solutions also were useful, in that they remind us that the challenges we face in GH development are substantial and at times may seem overwhelming, but that they are not unique to LMIC, and that we are not alone in facing the challenges.

There are several possible limitations to the study that should be mentioned. First, an online platform was used to conduct the assessment because of the challenges that postal surveys present for large international surveys [21], and because online surveys allow for logical branching (e.g., highlighting the barriers designated as most serious, for more detailed ratings). Web-based surveys sometimes have been criticized for low response rates, and for producing biased samples not representative of the target population. However, our response rate was comparable to that of many postal surveys and better than many online surveys [22], likely at least in part due to the specialized nature of the survey and the individualized invitation (i.e., each email was addressed with the specific name of the recipient). Further, because participants were individually selected and invited based on professional experience and provided with a restricted url (i.e., the url was not published or distributed generally), it seems unlikely that problems associated with sample self-selection with an open URL significantly influenced our sample beyond the general impact in any sampling where potential respondents have the right to decline to participate.

In addition, most of the significant effects in this study were relatively small, which does suggest that these barriers are relatively universal (i.e., vary relatively little across global health domain, etc.). However, even small effects can be important[23], when they are related to difficult to influence outcomes (e.g., global health barriers), or when effects are linked to major outcomes (in global health development, its budgets) and the importance of the questions it addresses (e.g., human mortality). Finally, it is important to note that this study assessed global health professionals’ perceptions of barriers, not objective indices of barriers (e.g., national per capita health care expenditures, as a resource limitation barrier). However, perceptions are critical in their own right, given human behavior and decision-making is largely based on perception [24].

In conclusion, we found that *Resource Limitations*, and *Priority Selection* barriers were seen as the most serious barriers whereas *Corruption*, *Lack of Competence*, and *Social and Cultural* barriers were seen as somewhat less serious. This provides some broad guidance for agency planning with, for instance, increased discussion of priorities between non-LMIC based administrators and professionals in the LMIC being useful. This latter point is reinforced by the finding that the *LMIC-oriented* and *Academic Global Health Achievement* variables operated in opposite directions. Overall, with a few exceptions, barrier seriousness did not differ greatly as a function of type of organization, health domain, or geographic region. This suggests that these barriers may be relatively fundamental at the system-level. The few significant effects may be important, however, for policy purposes. For instance, the fact that the Mental Health and HIV domains were seen as facing higher levels of social and cultural barriers suggests that it might be useful to emphasize such barriers at the planning and policy level for development in these areas. The individual-level effects we found (for *LMIC-oriented*, and *Academic Global Health Achievement*) highlight the importance of having a broad global health team that genuinely integrates the perspectives of different members; the individual-level effects also highlight the importance of taking an evidence-based approach to addressing barriers, in addition to taking an evidence-based approach to health care. Finally, it is hoped that the 22 suggested solutions, although not necessarily ‘innovative’, may provide GH professionals around with world with at least some new ideas for addressing the barriers within their own LMIC work.
